# Core Payload of the Space Gravitational Wave Observatory: Inertial Sensor and Its Critical Technologies

**DOI:** 10.3390/s24237685

**Published:** 2024-11-30

**Authors:** Shaoxin Wang, Dongxu Liu, Xuan Zhan, Peng Dong, Jia Shen, Juan Wang, Ruihong Gao, Weichuan Guo, Peng Xu, Keqi Qi, Ziren Luo

**Affiliations:** 1 Center for Gravitational Wave Experiment, National Microgravity Laboratory, Institute of Mechanics, Chinese Academy of Sciences, Beijing 100190, China; wangshaoxin@imech.ac.cn (S.W.); xupeng@imech.ac.cn (P.X.); 2 Taiji Laboratory for Gravitational Wave Universe (Beijing/Hangzhou), University of Chinese Academy of Sciences (UCAS), Beijing 100049, China; 3 Department of Modern Mechanics, School of Engineering Science, University of Science and Technology of China, Hefei 230026, China; 4 University of Chinese Academy of Sciences, Beijing 100049, China; 5 Hangzhou Institute for Advanced Study, University of Chinese Academy of Sciences (UCAS), Hangzhou 310024, China

**Keywords:** gravitational wave, space gravitational wave detection, inertial sensor, test mass

## Abstract

Since Einstein’s prediction regarding the existence of gravitational waves was directly verified by the ground-based detector Advanced LIGO, research on gravitational wave detection has garnered increasing attention. To overcome limitations imposed by ground vibrations and interference at arm’s length, a space-based gravitational wave detection initiative was proposed, which focuses on analyzing a large number of waves within the frequency range below 1 Hz. Due to the weak signal intensity, the TMs must move along their geodesic orbit with a residual acceleration less than 10^−15^ m/s^2^/Hz^1/2^. Consequently, the core payload-inertial sensor was designed to shield against stray force noise while maintaining the high-precision motion of the test mass through a drag-free control system, providing an ultra-stable inertial reference for laser interferometry. To meet these requirements, the inertial sensor integrates a series of unit settings and innovative designs, involving numerous subsystems and technologies. This article provides a comprehensive overview of these critical technologies used in the development of inertial sensors for space gravitational wave detection and discusses future trends and potential applications for these sensors.

## 1. Introduction

In 1915, Einstein proposed his general theory of relativity, which describes gravity as a property of space-time shaped by the distribution of mass and energy [1]. The presence of matter and energy causes space-time to curve, thereby influencing the motion of objects. As a result, trajectories that would be straight under Newtonian mechanics become curved geodesics.

This interaction between matter, energy, and space-time implies that any mass, under certain conditions, can generate self-sustaining gravitational waves that carry energy and propagate at the speed of light. These waves cause space-time to be squeezed or stretched as they move through it. Gravitational wave sources are abundant throughout the universe, arising from various celestial phenomena and providing valuable insights into the structure of astronomical objects [2].

Unlike electromagnetic radiation, gravitational waves interact very weakly with matter, allowing them to pass through most media with little attenuation. As a result, gravitational waves are extremely difficult to detect, and their effects can only be observed under extreme physical conditions, making their detection an exceptionally challenging task.

### 1.1. Gravitational Waves Detection on Ground

In the 1960s, Weber proposed the use of resonant rods to detect gravitational waves, a concept that initially faced considerable skepticism but nonetheless garnered significant attention and spurred early gravitational wave detection experiments [3]. By 1974, the discovery of the PSR1913+16 binary pulsar and the observation of its orbital period decay provided indirect evidence for the existence of gravitational waves [4]. Hulse and Taylor were awarded the Nobel Prize in Physics in 1993 for this groundbreaking work.

Around the same time as Weber, another gravitational wave detection method based on laser interferometry was proposed [5]. Offering a higher sensitivity, this technique gradually became the preferred approach for various gravitational wave detectors. A series of well-known detectors, including LIGO [6], VIRGO [7], GEO600 [8], and KAGRA [9], were established, forming a cooperative detection network to observe gravitational wave signals above a few Hz. In 2015, the advanced LIGO detector made the first direct detection of gravitational waves [10], and three leading scientists were awarded the Nobel Prize in Physics in 2017 for their outstanding contributions. Since then, LIGO and VIRGO have reported several gravitational wave events [11,12], opening a new window for cosmic observation and adding more detail to the rich tapestry of the universe.

### 1.2. Gravitational Waves Detection in Space

Due to seismic noise and the limited length of the interferometer arm, ground-based detectors primarily detect gravitational waves in the frequency range from 10 Hz to 1 kHz. This limitation makes it difficult to detect low-frequency gravitational waves, which are associated with a broader array of cosmic phenomena. In contrast, the space environment offers greater stability, allowing for longer interferometer arms capable of detecting gravitational waves below 1 Hz. The concept for a space-based gravitational wave detector was first proposed in the 1970s, envisioning the deployment of an antenna in deep space to capture these low-frequency signals [13].

Space gravitational wave detection relies on accurately measuring the displacement between two free-falling TMs as a gravitational wave passes through, thus resolving the signal. As a result, reference stability and measurement precision are critical. The system relies on two key subsystems: the noise shielding system (known as disturbance reduction system), which ensures an ultra-stable environment, and the laser interferometry system, which precisely measures any changes in distance between the TMs [14].

In the 1990s, ESA and NASA jointly proposed the first space gravitational wave detection program, LISA, with the primary goal of detecting gravitational waves from massive black holes and galactic binaries in the frequency range of 10 mHz to 0.1 Hz [13]. LISA aims to deploy three million-kilometer-class, forming an equilateral triangle, with each arm connecting pairs of TMs in geodesic orbits. The system will utilize heterodyne interferometry systems to detect tiny changes between the TMs.

Following LISA, several countries have proposed a range of space gravitational wave detection programs targeting middle- and low-frequency waves. These include ALIA [15], BBO [16], DECIGO [17], ASTROD [18], Taiji [19,20], and TianQin [21,22]. All of these missions follow the satellite-formation scheme established by LISA and adopt heliocentric orbits, except for TianQin, which utilizes a geocentric orbit. Based on this foundation, Taiji has innovatively proposed using joint formations between observatories to enhance the accuracy of source parameter estimation [23].

Given the complexity of implementing space gravitational wave detectors, these missions were carried out in multiple phases. LISA successfully launched its technology-demonstrating satellite, LPF, at the end of 2015. This satellite completed comprehensive verification of key technologies necessary for the future LISA mission, which is scheduled for launch in 2035 [24]. This lays a solid foundation for subsequent space gravitational wave detection missions. Meanwhile, Taiji and TianQin launched their first-phase technology demonstrating satellites, “Taiji-1” [25,26] and “TianQin-1” [27,28], in 2019, verifying similar technologies in Earth orbit with slightly lower accuracy. Both missions plan to deploy constellations for gravitational wave detection in 2033 and 2035, respectively [28,29].

### 1.3. The Inertial Sensors

To achieve the pure free-fall motion of the TM, space gravitational wave detectors rely on a disturbance-reduction system to minimize residual acceleration from non-conservative forces. The inertial sensor, also referred to as the gravitational reference sensor by LISA, is the core component of the disturbance-reduction system. Its primary function is to provide a highly stable inertial reference for the laser interferometry system, with a focus on the measurement and control of the TM. Capacitance-based measurement methods are the preferred choice for inertial sensors due to its high precision, stable structure, and ease of implementation. They can simultaneously measure TMs displacement and control them using frequency division multiplexing technology. Such inertial sensors were successfully used in several space-based physics experiments, including Earth gravity field measurement [30], equivalence principle verification [31], and gravitational effect testing [32].

Research on the inertial sensors began in the 1950s, initially focusing on measuring weak acceleration in space and monitoring environmental interference with satellites. In 1964, the concept of drag-free controlled flight for spacecraft was proposed [33], which laid the foundation for subsequent advances in inertial sensor resolution. In 1975, ONERA developed the first high-precision inertial sensor, called CACTUS, based on a spherical TM, which was employed to determine the resistance of spacecraft [34]. Throughout the 1990s, ONERA developed several models, including ASTAR, STAR, GRADIO, and Super-STAR, based on cuboid TMs and capacitance sensing for multi-DOF electrostatic servo control [35]. These systems were successfully implemented in various geodetic missions, including CHAMP, GRACE, GOCE, and GRACE-FO [36], and the highest measurement resolution that can be achieved was 3 × 10^−12^ m/s^2^/Hz^1/2^. The Taiji-1 inertial sensor, developed based on the STAR configuration, integrated laser interferometry and micropropulsion control systems to verify comprehensive technologies for future space gravitational wave detectors [37]. Its final acceleration measurement resolution was 3 × 10^−9^ m/s^2^/Hz^1/2^. Additionally, ONERA developed a small inertial sensor, MicroSTAR, to meet the needs of future microsatellite missions [38].

**Figure 1:**
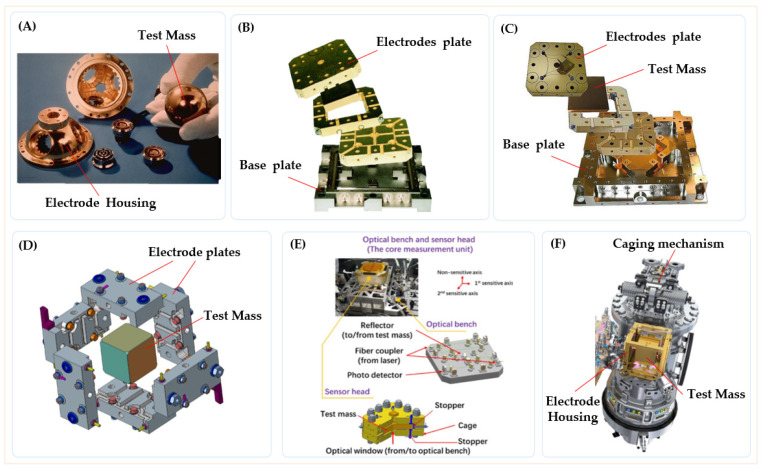
The structure of various types of space inertial sensor [37,38,40,41,42,43]. (**A**) Cactus accelerometer core; (**B**) ASTER sensor head; (**C**) Exploded view of GOCE accelerometer. (**D**) Mechanical core of the MicroSTAR accelerometer; (**E**) The optical bench and sensor head of Taiji-1; (**F**) Rendering model of one of the LPF’s inertial sensors.

Since the previous inertial sensors could not meet the requirements for space gravitational wave detection, ESA redesigned the inertial sensor for LISA using capacitive sensing technology, validating it as the primary target on the LPF mission. This inertial sensor is currently the only one with a complete functional structure, and many details in this article pertain to it. In-orbit results indicate that the acceleration noise reaches (3 ± 1) × 10^−15^ m/s^2^/Hz^1/2^ at 0.2 mHz, exceeding expectations [39]. The various inertial sensors described above are shown in Figure 1.

This article aims to provide a comprehensive introduction to the critical technologies used in inertial sensors, which serve as the core payload for space gravitational wave detection. Section 1 offers an overview of the background of space gravitational wave detection, as well as the development history of capacitance-based inertial sensor configurations. It emphasizes that the inertial sensor is one of the most complex and challenging components in space gravitational wave detection systems. Section 2 explains the basic principles of capacitive inertial sensors and outlines the primary noise constraints that affect system design. It also discusses the suppression methods employed to mitigate these noise sources. Section 3 delves into the key technologies associated with inertial sensors, exploring various technical solutions, with an emphasis on the application effects and continuity that these technologies provide. Finally, Section 4 analyzes the development trends of inertial sensors in space gravitational wave detection and explores potential application scenarios in other fields.

## 2. Working Principle and Design Constraints of the Inertial Sensor

For space gravitational wave detectors, the primary function of the inertial sensor is to provide a super-stable inertial reference. It shields the TM from disturbances and enables the TM to achieve free-fall motion along the sensitive axis, typically designated as the X-axis.

### 2.1. Basic Working Principle

**Figure 2:**
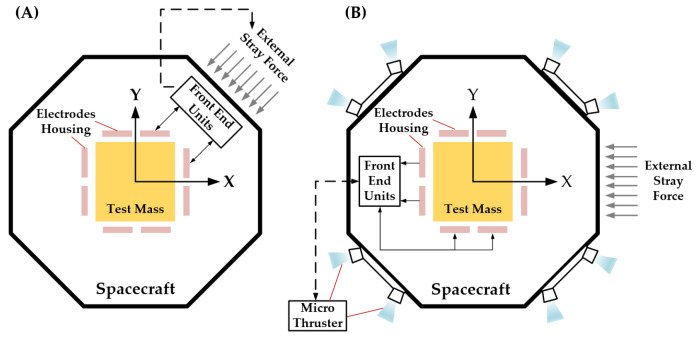
The basic working modes of the inertial sensor. (**A**) The TM in acceleration mode is located in the center of the electrodes housing; (**B**) The spacecraft follows the TM in science mode, achieving drag-free flight.

The inertial sensor is the core payload of space gravitational wave detection missions. Depending on the scientific objectives of the mission, two primary operating modes are typically designed, as shown in Figure 2: accelerometer mode and inertial reference mode.

**Accelerometer mode**: It is a calibration mode for the external disturbance of the spacecraft. These disturbances, which arise from non-conservative forces in the environment, result in relative displacement between the TM and the spacecraft. In this mode, the TM is controlled within a closed-loop system using multi-DOF electrostatic forces, allowing the TM to remain stably suspended at the center. The electrostatic force applied by the electrodes reflects both the magnitude and direction of external disturbances, which is why the inertial sensor is often referred to as an electrostatic suspension accelerometer.

**Inertial reference mode:** In space-based gravitational wave detection missions, each spacecraft is equipped with two TMs placed at a 60-degree angle relative to each other, with each TM located at a different interferometer arm. Achieving complete free-fall in all DOFs for both TMs is impractical. Therefore, it is sufficient to maintain free-fall along the sensitive axis, while the non-sensitive axes are controlled to keep the TMs close to their nominal positions. To maintain free-fall along the sensitive axis, the relative displacement of the TMs is measured by a capacitive sensor, which feeds data into the drag-free control system. Micro-thrusters are then used to compensate for non-conservative forces acting on the spacecraft, adjusting its position and orientation to ensure it follows the TMs during scientific measurements. Along the non-sensitive axes, electrostatic forces are applied in real time by the electrode plates to keep the TMs centered within their housing. This compensation prevents stray forces from introducing noise into the system. Since the inertial reference mode is crucial for scientific measurements, it is also known as scientific mode.

Additionally, in practical applications, the control strategy must be adjusted for different measurement axes, and the system must be able to switch between operating modes depending on experimental requirements [44].

**Figure 3:**
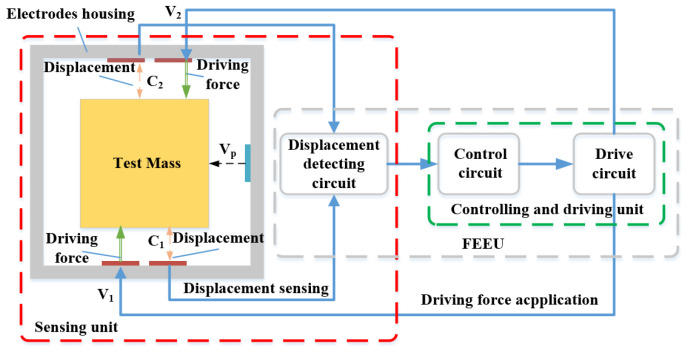
Basic functional structure of the inertial sensor. It mainly includes capacitive sensing and electrostatic drive control units.

Regardless of the operating mode—accelerometer or scientific—the inertial sensor must incorporate two essential functions: displacement sensing and motion control. These functions are achieved through a capacitive dynamic servo system, which utilizes predetermined electrode pairs to facilitate both displacement detection and feedback control of the inertial sensor relative to the TM, as illustrated in Figure 3. In the case of a cuboid TM, multiple independent control loops can be employed to ensure both position and orientation stability of the TM.

When considering capacitors *C*_1_ and *C*_2_, a displacement of Δ*d* results in a change in capacitance, denoted as Δ*C*:

(1) $${\Delta C=2\varepsilon }_{0}\varepsilon_{r}A\frac{\Delta d}{d_{0}^{2}{-\Delta d}^{2}}$$

In this context, $\varepsilon_{0}$ represents vacuum permittivity, $\varepsilon_{r}$ denotes relative permittivity, *A* is effective electrode area, *d*_0_ is initial spacing between the TM and electrodes, and *C*_0_ is the capacitance under this condition.

Utilizing a Taylor expansion of the capacitance formula for a more refined analysis:

(2) $${\Delta C=2\varepsilon }_{0}\varepsilon_{r}\frac{A}{d_{0}}\left[1+\frac{\Delta d}{d_{0}}+{\left(\frac{\Delta d}{d_{0}}\right)}^{2}+{\left(\frac{\Delta d}{d_{0}}\right)}^{3}+\ldots \right]$$

To minimize the impact of nonlinear errors and enhance measurement accuracy, the displacement of the TM (*y*) is typically designed to be very small. Under these conditions, it can be approximated as (*y* << *d*_0_):

(3) $$\frac{\Delta C}{\Delta d}\approx 2\frac{C_{0}}{d_{0}}$$

This approximation leads to a linear relationship between displacement and capacitance, enabling the determination of the position and direction of the TM by measuring the change in differential capacitance on both sides of the TM.

The motion of the TM is controlled by electrostatic feedback control. By measuring the capacitors *C*_1_ and *C*_2_ on either side of the TM and applying loading voltages *V*_1_ and *V*_2_ to the electrodes, the electrostatic force exerted on the TM is derived as follows:

(4) $$F=\frac{1}{2}{[\Delta C}_{1}\cdot {\left(V_{1}-V_{p}\right)}^{2}{+\Delta C}_{2}\cdot {\left(V_{2}-V_{p}\right)}^{2}]$$

where *V*_p_ is the preload voltage applied to the TM. If the circuit is strictly symmetrical, then:

(5) $${\Delta C}_{1}{=-\Delta C}_{2}{=\Delta C;\;V}_{1}{=-V}_{2}=V$$

When the TM is slightly displaced from its center position relative to the electrodes housing, the acceleration can be determined:

(6) $$a\approx \frac{2\cdot \Delta C\cdot V_{p}}{m}V$$

By utilizing this electrostatic force, a non-contact weak force can measure the control voltage applied to the electrodes, which in turn reflects the current acceleration and direction of the TM. This approach is particularly effective for capturing displacement changes within a small dynamic range, especially in microgravity environments, enabling ultra-high-resolution measurements.

### 2.2. Main Noise Constraints and Suppression Methods

Due to the strong correlation between the TM and the space environment, including the spacecraft and the front-end electronics unit, their interactions significantly influence high-precision free-fall motion. This, in turn, impacts the detection sensitivity of gravitational waves, potentially obscuring weak signals [45]. These multi-source, multi-physical field, and complex coupling interference factors represent fundamental constraints in the design of inertial sensors. Therefore, it is crucial to evaluate the proportion of acceleration noise and to mitigate its effects through various functional unit designs.

**Figure 4:**
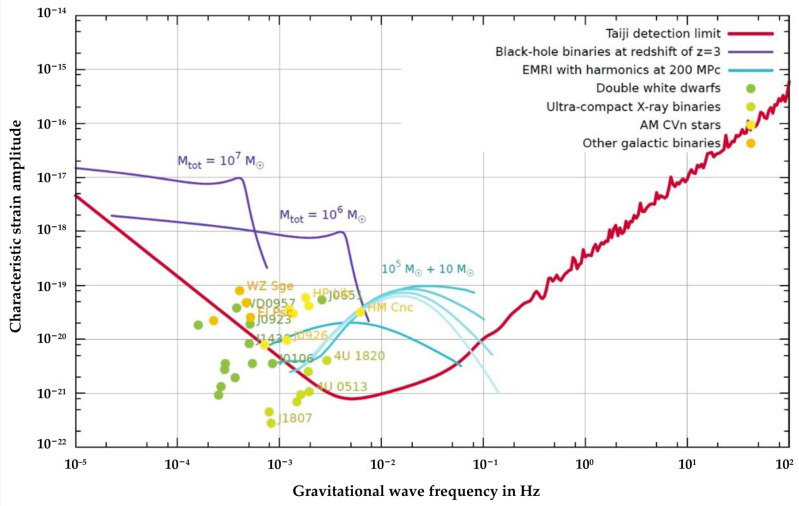
The sensitivity curve of the Taiji project [46]. Its main targets are supermassive black holes, intermediate-mass black holes, and double white dwarfs merge.

Taking the Taiji project as an example, the laser interferometer arm length *L* is about 3 × 10^6^ km. In the measurement frequency range from 0.1 mHz to 1 Hz, the gravitational wave signal with a strain amplitude spectral density *h’* = 10^−21^/Hz^1/2^ induces an optical path change *δL’* = *Lh’*~3 pm/Hz^1/2^, as shown in Figure 4. Consequently, the residual acceleration of the TM along the sensitive axis must meet the threshold of 3 × 10^−15^ m/s^2^/Hz^1/2^ [46].

The residual acceleration noise of the TM arises from two primary sources: 1. Direct disturbances affecting the TM; 2. Coupling disturbances that influence both the spacecraft and the TM. There are numerous known acceleration noise sources related to the inertial sensor, but only a few directly affect measurements and require specialized suppression technologies [47,48]. The following sections outline the main noise sources that significantly contribute to the overall system, along with their design constraints on inertial sensors. Additionally, current mainstream suppression methods for different noise types are discussed.

**Magnetic noise:** Fluctuations in both interstellar and local magnetic fields, along with magnetic field gradients, can induce magnetic moments, leading to significant disturbing forces. Additionally, if the TM becomes charged, the magnetic field can generate further disturbing forces via the eddy current effect.

Magnetic field noise is a primary source of interference in inertial sensors [49,50]. This type of noise involves multiple factors that must be carefully considered. The most effective methods for mitigating magnetic noise include minimizing the use of ferromagnetic materials and placing devices containing electromagnetic components at a distance from the sensor [51,52].

**Thermal noise:** Thermal gradients produce stray forces through various thermal phenomena. A major contributor is the radiometer effect caused by residual gas and temperature imbalances due to the interaction of gas molecules. Radiation pressure asymmetry also has a minor influence [53].

Thermal noise significantly impacts on system measurement, especially for low-frequency bands. While thermal gradients can be minimized by selecting materials with high thermal conductivity, the most effective strategy is to regulate ambient temperature through satellite-based temperature regulation [54].

**Brownian noise:** Brown noise arises from damping effects caused by molecular flow dissipation in the narrow gap between the TM and the surrounding electrodes housing [55]. A key feature of this noise is its frequency independence [56], meaning it must be strictly controlled across the entire operational range. Additionally, Brownian noise is temperature-dependent: as the ambient temperature increases, molecular thermal motion intensifies, leading to an increase in Brownian noise.

To mitigate Brownian noise, the TM must be placed in an ultra-high vacuum environment and operated within controlled temperature conditions.

**Charge noise:** Cosmic rays and high-energy particles can cause the TM to accumulate charge, leading to electronic noise. As charge continues to accumulate, the noise level increases. Therefore, effective charge control is necessary to prevent further noise buildup.

To manage charge accumulation without introducing additional noise, the inertial sensor is equipped with a charge management system that neutralizes the charge on the TM via photoelectric discharge [57].

**Drive voltage noise:** The inertial sensor applies force and torque to the TM using an electric field. However, the voltage used to create driving forces can be noisy, generating stray forces and torques. Geometric defects may also cause leakage of these forces. The stray noise generated by capacitance sensing in the inertial sensor and front-end electronics unit must be kept below than 1/10 of the sensitivity level of the TM acceleration [58].

To mitigate electronic noise, the design must minimize noise generation, and the manufacturing quality of the capacitive sensor hardware must be enhanced.

**Coupling noise:** In addition to directly acting noise, coupling noise can occur when noise from non-sensitive directions inadvertently transfers to sensitive directions, causing acceleration noise in the TM. Two primary factors contribute to this: gravitational gradients and capacitance gradients, with the gravitational gradient having a more pronounced effect [46,59,60].

The capacitance gradient stems from potential fluctuations due to variations in work function across different positions on the metal surface, often caused by surface coating non-uniformity and contamination. Therefore, it is crucial to strictly control the cleanliness of the sensor hardware and use high-power function materials to manufacture. Moreover, a peripheral monitoring system must be established to accurately predict the ambient conditions around the test mass. A torsion weighing device can be used to verify noise models.

Noise remains the primary challenge restricting the development of inertial sensor, which involves almost all aspects of inertial sensor, so its control is also a complicated process, which needs comprehensive consideration. In addition, it is also necessary to set up a peripheral monitoring system to accurately predict the ambient conditions around the test quality and build a torsion-weighing device to verify the corresponding noise model, which provides a sufficient basis for further optimizing the system design under noise constraints. Thanks to the successful launch of the LPF, many noise models and their suppression methods have been validated in orbit, yielding results that exceeded expectations. Consequently, most of these measures will continue to be employed in future missions.

## 3. The Critical Technologies of Inertial Sensor Development

It has been repeatedly emphasized that gravitational waves can be detected only if the TM follows a geodesic trajectory [61]. As such, the primary challenge in space-based gravitational wave detection is ensuring that the TM moves along the geodesic with high precision. This is why inertial sensor is used as the primary space gravitational wave-detection device: it provides the TM with protection from external disturbances, enabling it to serve as an extremely accurate inertial reference for the laser interferometry system.

### 3.1. The Composition of Inertial Sensor

**Figure 5:**
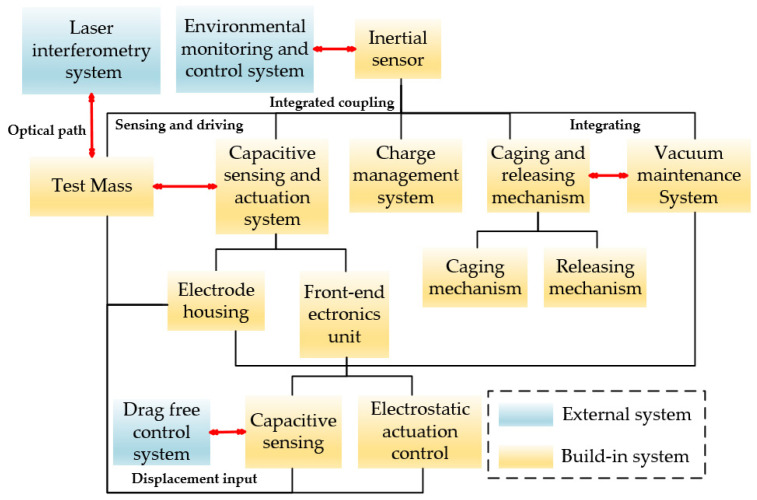
The basic composition and the relationship network with the external system of the inertial sensor. It contains most of the critical technologies and relationships.

As discussed in Section 2, acceleration noise presents a fundamental limitation in the development of inertial sensors. In addition to noise suppression, several engineering considerations must be addressed, including size constraints, weight, power consumption, cleanliness, vibration and shock during launch, vacuum maintenance, and integration with external systems [62,63]. To overcome these challenges, various subsystems and units with distinct functionalities have been incorporated into the inertial sensor design, as illustrated in Figure 5. The following sections will provide a detailed overview of the key techniques employed in the development of these sensors.

### 3.2. The Test Mass

In the current phase of space gravitational wave detection missions utilizing laser interferometry, each spacecraft is equipped with two TMs that serve dual purposes: acting as terminal reflection mirrors for the interferometer and functioning as inertial reference elements for detection. These TMs are influenced by a variety of constraints and interferences stemming from a complex multi-physical field environment, leading to significant noise contributions from the TMs themselves [48]. The cubic TM design offers clear advantages over spherical alternatives, particularly in terms of manufacturing and control, enabling better decoupling between axes. As a result, cubic TMs have become the preferred design for modern gravitational wave-detection missions [62].

To suppress noise at its source, two primary strategies can be employed: increasing the mass of the TM, which inherently reduces its sensitivity to external disturbances, and improving the manufacturing quality of the TM to lower coupling errors. Additionally, it is essential to mitigate gravitational variations between the spacecraft and the TM.

Material selection plays a pivotal role in noise reduction and should be prioritized. Alloys with low magnetic susceptibility, such as TC4, Au/Pt, Pt/Rh, Au/Ir, and Ag/Cu, are commonly utilized to create ultra-low-disturbance TMs with high density [64,65,66]. Maximizing the TM’s inertia while minimizing both internal and external magnetic disturbances is key to reduce acceleration from stray forces.

Additionally, potential defects or contamination during the manufacturing process of TMs—such as charge accumulation, patch fields, and magnetization—must be carefully controlled. Solutions include surface gilding, maintaining cleanliness, and implementing demagnetization or the structural strain regulation techniques [67].

**Figure 6:**
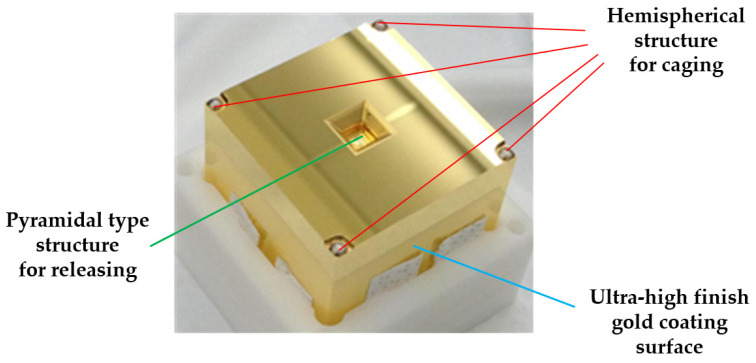
The test mass of LPF [43]. Contains structural features for caging and releasing.

For the LISA mission, the selected TM material is an Au–Pt alloy with a mass ratio of 73:27. This choice results in a magnetic permeability of |*χ*| < 10^−5^ and a residual magnetic moment of *m*_0_ < 10^−8^ Am^2^ [68], ensuring high density while allowing for a single-phase alloy formation. The TM is designed as a 46 mm cube with a mass of 1.96 kg. High-precision optical machining and finishing processes are applied to achieve tight geometric tolerances and high reflectivity, significantly reducing coupling noise. The entire surface of the TM is gold-coated to ensure uniformity and consistent potential measurement under electric fields. Additionally, an interface for the caging and releasing mechanism is also included, facilitating operational functionality, as depicted in Figure 6.

### 3.3. Capacitive Sensing and Electrostatic Actuation System

In any operating mode, the inertial sensor must continuously monitor the pose status of the TM in real time, while also driving and controlling its motion. This process is achieved through a capacitive sensing and electrostatic actuation system, which consists of two key components: the electrode housing and the front-end electronics unit. The electrode housing is responsible for creating an array of capacitive sensors around each test mass, while the front-end electronics unit detects and controls all DOF of each TM through electrodes embedded within the housing [69].

In addition to addressing challenges such as magnetic field variations, temperature gradients, and electrostatic field crosstalk caused by the surrounding environment, the most critical issue for the capacitive sensing and electrostatic drive system is achieving ultra-high precision in the capacitive sensors and electrostatic drive control. The following sections provide a detailed discussion of these technical challenges.

#### 3.3.1. Electrode Housing

Capacitive sensing and electrostatic drive control depend on the electrode housing, which is the component closest to the TM and operates in the same environment. To minimize the influence of noise—such as magnetic fields, temperature gradients, and axial coupling—the electrode housing must be fabricated from materials with low magnetic susceptibility, high thermal conductivity, low thermal expansion, and good machinability. Additionally, the gap between the electrodes and the TM should be maximized to reduce the impact of stray forces and noise. To ensure a uniform electric field around the TM, the electrode housing should be coated with a material that has a high work function. The guard rings should also be placed between the electrodes to mitigate the electric field edge effect.

**Figure 7:**
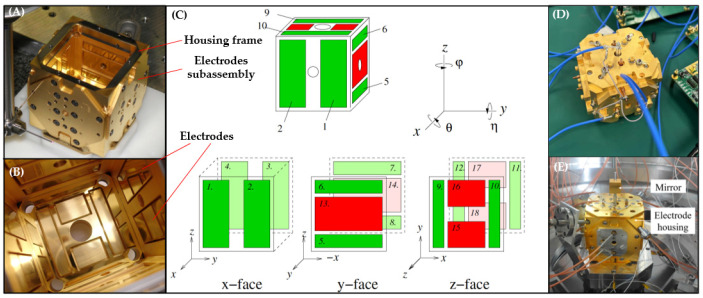
Electrode housings for different space missions [58,70,71,72]. (**A**) LPF electrode housing being tested; (**B**) Internal structure of LPF electrode housing; (**C**) Electrodes division of LPF; green indicates the sensing and driving electrodes, and red indicates the injection electrodes; (**D**) The electrode housing of Taiji; (**E**) The electrode housing of TianQin.

The LPF proposes a classical configuration for the electrode housing, based on an electrostatic suspension accelerometer. This configuration consists of 18 electrodes, as illustrated in Figure 7A–C. The red electrodes are injection electrodes used to polarize the TM with an AC voltage, while the green electrodes serve as sensing electrodes to detect the TM’s position and to apply driving forces (control and capture) acting on the TM.

For the LPF mission, the gaps between the electrodes and the TM are designed to be 4 mm, 3.5 mm, and 2.9 mm, respectively—larger than those used in other non-drag space missions. This design effectively mitigates stray force noise, particularly the patch field effect. In parallel, considering stiffness and thermal effects, multiple technical iterations were conducted before selecting molybdenum (Mo) as the material for the electrodes housing structure. Mo demonstrated favorable qualities in terms mentioned above. All the surfaces are gold-coated. Additionally, Taiji and TianQin created matching electrode housings for testing based on the LPF structural design, as shown in Figure 7D,E.

#### 3.3.2. Capacitive Sensing

Capacitive sensing is a precise displacement measurement technology widely used in space missions. This technology works through a collaboration between the electrode housing and the front-end electronics unit. The electrodes housing, firmly attached to the spacecraft, forms a pair of differential capacitors with electrodes that face the TM. As described in Section 2.1, when the TM is displaced, the capacitance values on both sides change accordingly. By measuring the change in differential capacitance through the front-end electronics unit, the relative displacement of the TM relative to the electrodes housing can be accurately determined, thus defining the pose state of the TM.

**Figure 8:**
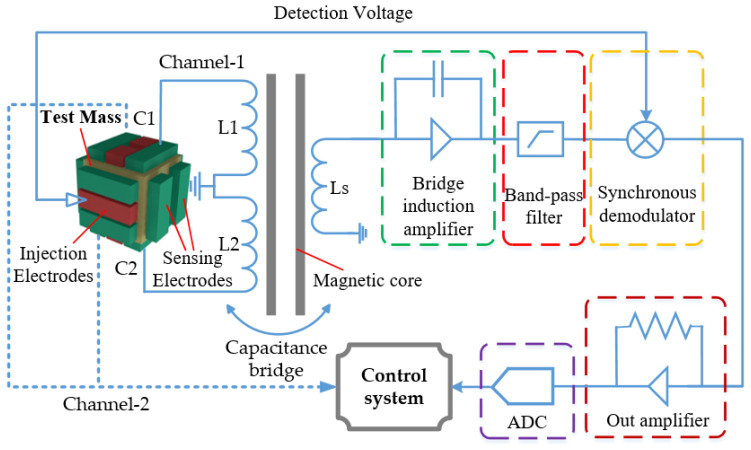
Schematic diagram of the capacitive sensing system.

The induction bridge circuit plays a critical role in enabling capacitive sensing technology [73]. Its basic principle is illustrated in Figure 8. The front-end electronics unit connects directly to the electrodes via shielded wiring. Capacitive elements (*C*_1_, *C*_2_) and inductive elements (*L*_1_, *L*_2_) are selected to resonate at a specific frequency. An excitation signal matching this frequency is applied to the TM through injection electrodes, typically arranged along non-sensitive axes. As the TM moves, it induces a current imbalance between *L*_1_ and *L*_2_, generating a differential voltage across the secondary winding (*L*_s_) of the transformer formed by *L*_1_ and *L*_2_. This weak voltage is amplified by a bridge induction amplifier and is further boosted by a high-frequency amplifier, preserving its frequency and waveform. Components matching the excitation signal frequency are extracted through band-pass filtering, followed by synchronous demodulation. The analog signal is then converted into a digital signal by an analog-to-digital converter. Finally, the displacement data is processed and transmitted to a computer, which feeds it into the drag-free control system. This system sends ignition commands to the micro-thruster, enabling the spacecraft to track the TM and establish optimal conditions for scientific measurements.

Each channel of the inertial sensor is equipped with a bridge sensing circuit, as described above. This allows for the determination of both translational and rotational displacements of the TM. This capability enables the identification of the DOF state of the TM under various operational conditions. When the sensor operates in scientific mode with high sensitivity, its range is limited to approximately 100 μm, and the corresponding capacitive sensing noise is about 1 aF/Hz^1/2^(10^−18^ F/Hz^1/2^). This translates to a displacement sensitivity of roughly 1~2 nm/Hz^1/2^, and a rotation sensitivity of 80 nrad/Hz^1/2^, primarily influenced by background noise [74].

One of the main challenges of capacitive sensing is the suppression of low-frequency electronic noise. Through the careful selection of components and circuit optimization, the Swiss Federal Institute of Technology Zurich developed a capacitive sensing circuit with a resolution of 1 × 10^−7^ pF/Hz^1/2^ resolution (above 10 mHz) for the LISA mission [58]. This circuit was thoroughly validated during the LPF mission. On-orbit experiments demonstrated that the system’s sensitivity approaches the thermal noise limit of the transformer [75], confirming that background noise from the bridge is a major source of capacitance-sensing errors [47]. To mitigate these noise issues, TianQin developed a transformer with a high-quality factor to reduce the current noise from the preamplifier. As a result, the capacitance noise above 1 mHz improved to better than 3 × 10^−7^ pF/Hz^1/2^, corresponding to a TM displacement noise of less than 0.74 nm/Hz^1/2^ [76,77].

#### 3.3.3. Electrostatic Actuation Control

**Figure 9:**
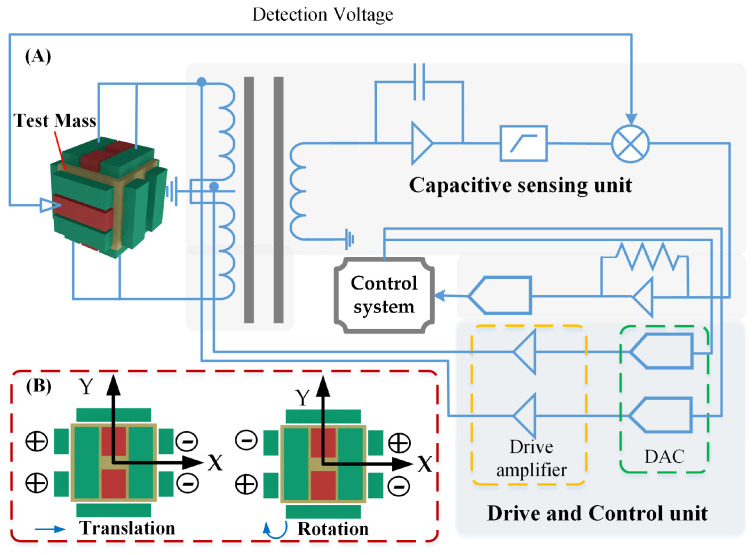
The basic principle of electrostatic drive and control in single DOF [58]. (**A**) Basic components of electronic circuits; (**B**) Voltage configuration controls translation and rotation of the TM.

In addition to measuring the displacement of the TM, a key function of the capacitive sensor is to drive and control the TM through electrostatic forces, as discussed in Section 2.1. The TM can only achieve free-fall in the sensitive axis direction. In the non-sensitive axis direction, however, it may drift slowly relative to the spacecraft due to external forces. This drift cannot be adjusted by microthrusters, requiring active control of the TM. The positional relationship between the spacecraft and the TM is continuously adjusted, with the block diagram of its structure shown in Figure 9A.

The inertial sensor utilizes frequency-division multiplexing technology. Based on the mechanical configuration of the electrode housing, the drive circuits and sensing circuits share the primary winding and electrodes of the transformer. Twelve sensing electrodes are used simultaneously for both sensing and driving, thereby enhancing system integration. By utilizing the displacement information of the TM obtained from the capacitive sensing unit and combining it with feedback control loop requirements, a feedback voltage is applied to the corresponding electrode, generating the necessary electrostatic force or torque to control the translation or rotation of the TM, as illustrated in Figure 9B.

The primary challenges in electrostatic drive and control technology are the operational range and drive voltage noise. The operational range refers to the various functions of the inertial sensor under different working conditions, such as capturing the TM upon its release into orbit. The required driving force is on the order of nanometers, necessitating precise control of the TM in drag-free conditions. To meet high-resolution requirements, the maximum driving force of the system is typically limited to only a few μN [78]. Voltage noise significantly impacts the control accuracy of the TM. The LISA team has achieved amplitude stability of 3~8 ppm/Hz^1/2^ through extensive ground experiments. Higher voltage levels tend to increase noise, which adversely affects the TM. Therefore, the electrostatic drive system primarily relies on AC voltage control, which effectively decouples different DOF using sine waves of varying frequencies [63]. Additionally, PID or PWM techniques are employed to achieve high-precision drive control of the TM.

#### 3.3.4. Electrostatic Coupling Compensation

Electrostatic drive technology fulfills two primary functions: It provides an amplitude-modulated carrier for force driving and generates a low-frequency AC voltage for detecting the charge of the TM [58]. However, any changes or gradients in the electric field associated with the motion of the TM can couple it to the electrode housing and, ultimately, to the spacecraft. Thus, another critical function of the capacitive sensing and electrostatic actuation system is to compensate for coupling effects arising from various noise sources.

As outlined in Section 2.2, electric field-related coupling noise primarily stems from two sources. The first is charge accumulation, which requires regulation by a specialized charge management system—this will be covered in subsequent chapters. This chapter focuses on the second source: patch field effects.

Patch field effects arise from the uneven distribution of surface potential due to spatial and temporal variations in the work function of the TM material. These variations give rise to stray DC electric fields, which can couple with the fluctuating charge on the surrounding electrode housing, thereby generating sensing and actuation noises. Patch field effects represent a significant noise source in inertial sensors [47]. These effects are primarily linked to the surface condition of the materials. For this reason, TM and electrode housings are typically gold-coated, owing to gold’s high work function. Additionally, maintaining a clean environment during manufacturing and integration is essential.

To accurately compensate for coupling noise, it is essential to quantify the magnitude of the patch field effect. This can be achieved through several techniques, including the use of a Kelvin probe, a dedicated torsion pendulum apparatus, and detailed electric field modeling of the surrounding structure of the TM [79,80]. The Kelvin probe, while effective, is a contact-based measurement technique that can be risky for high-precision surfaces, and it is therefore rarely used in this context [81]. In contrast, the torsion pendulum utilizes an electronic unit to measure the changing potential difference, thereby reflecting the magnitude of the patch field effect [82].

The University of Washington has developed a simple device to obtain time-distribution data of potential differences, with a measurement sensitivity of 30 μV/Hz^1/2^@0.1 mHz [83]. Huazhong University of Science and Technology further developed a spatial scanning probe and introduced a dual-frequency mode signal injection method, which improved measurement efficiency. The resolution is expected to reach 2–4 μV/Hz [84]. Meanwhile, the University of Trento has developed a torsion pendulum based on a prototype principle that simulates sinusoidal charge variations on the TM by applying a jitter voltage to selected electrodes. This device has been experimentally validated [85]. Experimental results show that applying a DC compensation voltage can reduce the average bias voltage caused by changes in the work function by approximately 100 times, significantly lowering the corresponding acceleration noise to negligible levels. Furthermore, a charge management system can be integrated with the torsion pendulum to control the test mass charge, further improving measurement accuracy [86].

The most effective solution, however, is to conduct on-orbit testing and calibration. This would involve using the front-end electronics and charge management system for joint measurement. Additionally, it may be possible to predict patch field effects statistically by constructing specific physical models, although this approach has yet to be experimentally confirmed [87].

In addition to compensating for patch field effects, other coupling effects are mitigated by adding a small DC voltage to the electrodes. This helps offset potential deviations, achieving effective compensation for coupling noise.

### 3.4. Charge Management System

In space gravitational wave detection, most non-conservative forces from space are isolated by the spacecraft and the multi-layer structure of the inertial sensor. However, high-energy particles and radiation can still penetrate the spacecraft shell and reach the TM surface, leading to charge accumulation [88,89]. As discussed in Section 2.2, this charge buildup introduces significant noise, making the measurement and control of the TM charge critical for space gravitational wave detection. This is typically achieved through a charge management system [90].

Several space missions, such as CHAMP, GRACE, and GOCE [36], have encountered charge accumulation problems. These missions typically employ gold wire connections to continuously dissipate accumulated charge, effectively reducing noise. While this method is simple and effective, the direct contact approach introduces mechanical noise from the conductor, even when μm diameter gold wires are used. As a result, the noise level is approximately 10^−13^ m/s^2^/Hz^1/2^ [91,92], which is several orders of magnitude higher than the requirements for inertial sensors. As a result, contact-based methods are unsuitable for space gravitational wave detection. Therefore, a non-contact approach is necessary to achieve precise control over the TM charge [93].

The non-contact method for charge management utilizes the photoelectric effect, where laser photons strike a metal surface, generating photoelectrons. These photoelectrons neutralize charge accumulation on the TM surface, effectively managing its charge. UV photons are particularly effective in this process due to their higher energy, making UV light the preferred choice for charge management systems in inertial sensors [94].

**Figure 10:**
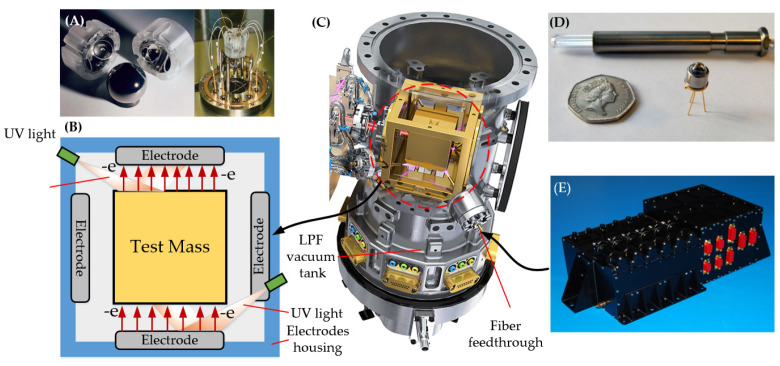
The composition and principle of a charge management system [96,102]. (**A**) The gyroscope of GP-B in which the charge management systems are integrated; (**B**) Schematic diagram of excitation charge in different regions of Laser radiation; (**C**) Rendering of the LPF inertial sensor; (**D**) Size comparison of the LED and mercury lamp; (**E**) The UV source of LPF.

Unlike conductive gold wire, the non-contact UV discharge method provides charge control without introducing mechanical noise. However, its complexity presents challenges in implementation. This technology was initially developed at Stanford University and has been successfully applied in the GP-B mission [95]. It is now considered a leading technology for charge management in high-precision inertial sensors. Imperial College designed a charge management system for the LPF based on this approach [96]. This system uses three fiber optic guide units within a vacuum enclosure to irradiate the electrode housing and TM. When UV light strikes the TM or electrodes, the photons transfer their energy to electrons in the material. If the energy exceeds the material’s work function, electrons are ejected from the surface as photoelectrons. These photoelectrons combine with the positive charge on the TM, neutralizing the charge and adjusting the TM’s charge state [97]. Both GP-B and LPF employed RF mercury lamps as light sources, introduced into the inertial sensor via a fiber flange, as illustrated in Figure 10.

Despite their effectiveness, RF mercury lamps have several limitations, including long warm-up times, limited dynamic ranges, short lifespans, and susceptibility to RF and electromagnetic interference. Additionally, positioning the light source within the system design presents challenges. An alternative to RF mercury lamps is the use of ultraviolet light generated by semiconductor devices such as UV LEDs. Many research institutes are investigating the reliability, stability, lifespan, charge and discharge efficiency, and charge management strategy, and experimental devices are being developed [98,99,100,101]. UV LEDs offer several advantages over mercury lamps, including smaller size, lighter weight, lower power consumption, and easier integration. They can also be managed outside the gravitational wave signal band using AC charge transfer technology, minimizing interference with scientific measurements. Furthermore, UV LEDs provide the ability to adjust intensity, pulse width, and pulse frequency to meet dynamic range requirements, positioning them as a promising solution for future charge management systems in inertial sensors.

### 3.5. Caging and Releasing Mechanism

Spacecraft are subject to atmospheric vibrations and impacts during launch, which expose internal payloads to extreme mechanical conditions. To address these challenges, many space systems integrate additional mechanisms for in-orbit locking and unlocking [103,104]. Examples include satellite-rocket connections [105], space robotic arms [106], and expandable array antennas [107]. Unlike precision instruments, these mechanisms are designed with flexibility in mind and utilize components such as crank sliders, gears, cams, nuts, and pressure rods. They are typically driven by devices like motors, fuses, paraffin, shape memory alloys, or electromagnets to achieve their intended functions.

**Figure 11:**
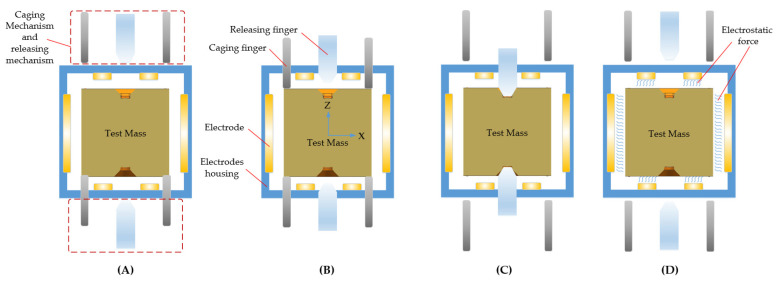
Basic workflow of the caging and releasing mechanism. (**A**) Initial state, the TM sits on the caging fingers; (**B**) Caging state, the TM is locked by the caging fingers; (**C**) Caging to position state, the TM is transferred from the caging mechanism to the releasing mechanism; (**D**) Releasing state, the TM is released and captured by electrostatic force.

In contrast, the non-contact state required between the TM and the electrodes—maintained at a significant gap—necessitates a specialized locking device. This device must mitigate the destructive forces during launch and ensure the safe positioning of the TM, which weighs approximately 2 kg, into its intended orbit. Once in orbit, the TM must be captured using electrostatic force; however, this force is limited to μN levels [108]. Thus, capturing the TM at an ultra-low residual speed solely through electrostatic force is crucial for entering scientific mode [109]. This presents a significant challenge, as the locking mechanism alone cannot achieve the ultra-low-speed release. Therefore, an additional set of releasing mechanisms must be integrated to function in conjunction with the locking mechanism. The functions of each mechanism are summarized in Table 1 below:

To achieve the functions of locking and releasing the TM in a specific sequence, a multilevel control strategy was designed, as illustrated in Figure 11. The basic operational process is as follows: 1. The TM rests on the bottom fingers; 2. The locking mechanism fully engages with a clamping force greater than 1000 N to cage the TM; 3. The release mechanism applies tens of newtons of force to locate and grab the TM while the caging mechanism retracts, completing the transition; 4. The releasing mechanism disengages the TM and returns, while electrostatic force captures the TM. The two driving mechanisms will be introduced separately in the following sections.

#### 3.5.1. Caging Mechanism

Section 3.1 provides an overview of the fundamental configuration of TMs used in space gravitational wave detection. Regardless of whether the TM is spherical or cubic, a clamping force is applied vertically at both ends to secure it in place.

**Figure 12:**
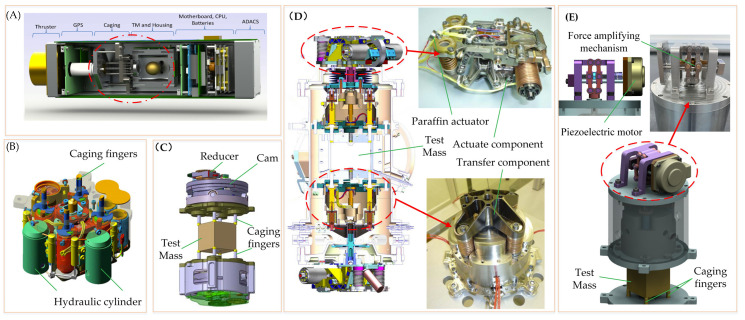
Different types of TM caging mechanisms [110,111,112,113,114,115]. (**A**) The mechanism designed by Stanford university for the spherical TM; (**B**) Hydraulic caging mechanism by Alenia Space Italy for LISA; (**C**) Cam and reducer caging mechanism designed by Astrium for LISA. (**D**) Paraffin motor and friction wheel caging mechanism by RUAG for LISA; (**E**) Piezoelectric motor and force amplification caging mechanism designed by Taiji.

For spherical TMs, Stanford University has designed a caging device that utilizes a motor-gear reduction system, inspired by the GP-B satellite technology, to lock the TM [111,112]. This device, shown in Figure 12A, has been tested in aero-level free-fall experiments.

For cubic TM, the clamping area is symmetrically positioned at the eight angular edges along the Z+ and Z− directions to maintain uniform force distribution and minimize deformation caused by clamping forces. Additionally, a hemispherical surface is designed for precise positioning and alignment, as shown in Figure 6. Based on this structural configuration, Thales Alenia Space Italy developed a caging mechanism for LISA [113], depicted in Figure 12B. This mechanism uses hydraulic pressure to drive locking rods, ensuring uniform preload distribution and providing a reliable lock on the TM.

At the same time, Astrium introduced an alternative caging mechanism based on a piezoelectric motor, gear reducer, and cam. Numerous experiments have been conducted with positive results. In this design, the output torque of the piezoelectric motor is amplified by the gear reducer, and the up-and-down motion of the ejector rod, generated by the cam mechanism, applies the locking force on the TM [114], as shown in Figure 12C.

Subsequently, RUAG designed a new locking mechanism for LISA [115], as shown in Figure 12D. This design features a paraffin actuator with slow output displacement, along with various components such as a cam, friction plate, multi-connecting rods, bel-lows, and springs, forming a complete mechanism. The system cages the TM on the ground, using friction brakes to prevent the cam from rotating. When the spacecraft reaches its intended orbit, the paraffin actuator activates, pushing the friction plate to release the compression springs, allowing the cam to rotate and slowly release the TM. To mitigate the impact of vibration and shock during operation, the cam curve has been specially optimized. Additionally, the system employs a combination of rigid and flexible springs with varying stiffness to achieve a uniform distribution of the caging force, while considering the exhaust function of the vacuum system, known as CVM. This scheme was ultimately adopted by the LPF, which successfully completed its scheduled on-orbit mission.

Building on the LPF caging device, the Taiji team simplified the actuator by proposing a structure that uses a piezoelectric motor drive and a diamond-shaped amplifier output, ensuring reliable locking of the TM. This new design was applied to the staged prototype, as shown in Figure 12E.

The complete workflow of the caging mechanism includes ground caging and space uncaging. Its function is relatively straightforward, requiring no repetitive operations in orbit, thus allowing flexibility in implementation based on mission characteristics and system design. However, since the locking mechanism cannot re-lock the TM once it has been unlocked, it is often considered a single point of failure, necessitating a high degree of reliability.

#### 3.5.2. Releasing Mechanism

According to Section 3.3.2, the design distance between the TM and the electrodes is only a few millimeters, resulting in a very small electrostatic control force on the TM. As a result, the TM must be released at a low speed (5 μm/s) with high precision (100 μm); otherwise, successful capture will not occur [116].

**Figure 13:**
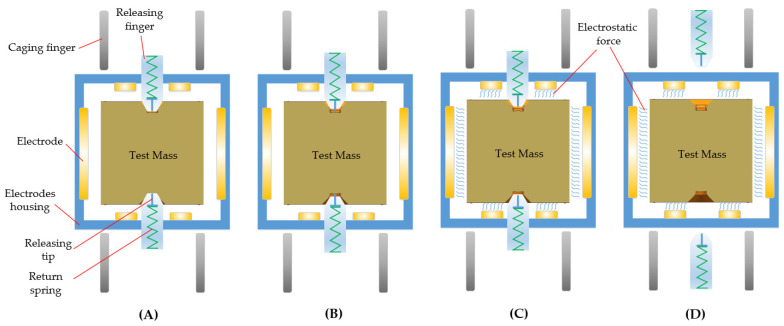
Operation strategy of the releasing mechanism. (**A**) Position state, the TM is held by the releasing fingers; (**B**) Holding state, the releasing tips elongate to contact the TM, and the releasing fingers slowly retract; (**C**) Releasing state, releasing tips quickly retract to release the TM; (**D**) Capturing state, the electrostatic force captures the TM, and all mechanisms return to their initial position.

To meet these stringent requirements for the releasing mechanism, a key challenge is minimizing the cold-welding effect between the mechanism and the TM in space conditions. The most straightforward approach involves reducing both preload and the interface contact area. However, this cannot be achieved with a single-stage mechanism, as shown in Figure 12D; instead, a multi-stage strategy must instead be employed. The basic workflow of this strategy is as follows: 1. The TM is grasped by the releasing fingers while the internal releasing tips remain retracted; 2. The tips move downward to make contact with the TM, and the fingers retract to decrease clamping force; 3. The tips quickly retract; 4. The fingers continue to retract, allowing the electrostatic force to capture the TM, as shown in Figure 13.

**Figure 14:**
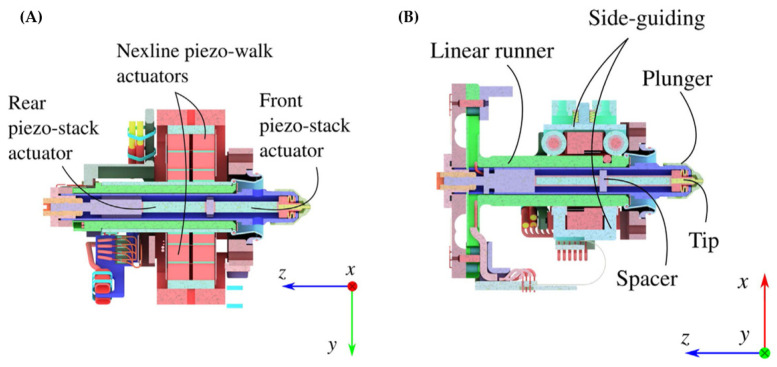
The composition structure of LPF GPRM in detail. (**A**) YZ-plane section shows the piezo actuator move the plunger along Z; (**B**) XZ-plane section illustrates the side-guiding system. The GPRM is distributed on both sides of the TM [117].

Initially, RUAG designed the releasing mechanism for LISA based on this strategy [117], which was implemented in the LPF mission. The mechanism utilized a high-precision inchworm piezoelectric actuator, Nxline^®^, to drive the fingers, while piezoelectric stacks and disc springs facilitated rapid expansion and retraction of the tips. The design is both ingenious and compact, leveraging the precise displacement output and quick recovery characteristics of piezoelectric ceramics. Additionally, it is equipped with guiding units, displacement sensors, force sensors, and other functional devices to ensure accuracy and reliability, as shown in Figure 14.

In contrast to the caging mechanism, the releasing mechanism may require multiple attempts to achieve the desired outcome. Therefore, it must demonstrate high stability and repeatability and necessitates robust ground verification testing to ensure its functionality. Given that the space environment differs significantly from terrestrial conditions, tribological issues become a primary concern for the space mechanism [118]. However, simulating the cold-welding effect on Earth is challenging, and the adhesive forces involved in the releasing mechanism are minimal. Consequently, a specialized verification device is essential [119].

To this end, Trento University developed a unique device to evaluate adhesion and conducted extensive ground tests, including system modeling [120,121,122], device construction [123,124], and statistical analysis [109,125]. By studying the adhesion force, it is anticipated that the on-orbit performance of the device can be reasonably predicted. The TianQin project also conducts ground testing of the TM release impulse using aluminum alloy based on the complex pendulum, which provides a feasible measurement scheme for pulse testing in engineering implementation [126].

Despite these analytical and experimental efforts to assess the releasing mechanism’s performance, the success rate of releases after the LPF launch was less than 30%. Although the work was completed through several re-injection procedures, confirming the correct implementation of the mechanism, various issues arose during the actual process due to symmetry errors and misalignment, and the release residual velocity was not zero. In response, the LPF team conducted further research on the control strategy in orbit, yielding positive results. They concluded that misalignment of the thimble and collisions between the fingers and the pyramid during the release process contributed to the challenges. They also proposed a comprehensive analytical method to explain the mechanism’s dynamic response, providing valuable insights for future unit designs in similar missions [127,128,129].

### 3.6. Vacuum Maintenance System

The analysis of residual acceleration noise from the inertial sensor highlights the significant impact of environmental factors, particularly Brownian noise from residual gas and radiometer effects due to temperature coupling [72]. Notably, Brownian noise can exceed the total system noise at low frequencies, as confirmed by LPF’s in-orbit experiments [130]. After nearly 2 years of outgassing, the vacuum around the TM stabilized at approximately 10^−5^ Pa [131], but further improvement to 10^−6^ Pa is necessary for LISA [132]. Therefore, the inertial sensor requires a vacuum maintenance system to ensure optimal performance.

Vacuum maintenance in space devices is typically achieved through several methods: getters to absorb residual gas, ion pumps to neutralize participating gases, and systems to discharge residual gas into cold space. Each method has its own advantages and disadvantages, with their use dependent on specific requirements. ONERA has extensive experience in developing electrostatic accelerometers, successfully using getters to maintain long-term vacuum conditions in orbit, achieving vacuums as low as 10^−9^ Pa [133]. In some instances, the operational current of micro-ion pumps has also been employed to monitor sensor vacuum levels. In the early stages of LISA, a getter solution was implemented. However, during the implementation phase of the LPF, an exhaust solution was ultimately chosen. This approach was cleverly integrated into the caging mechanism designed by RUAG [114], which included a valve that connected the inertial sensor to outer space, ensuring ultra-high vacuum conditions were maintained.

**Figure 15:**
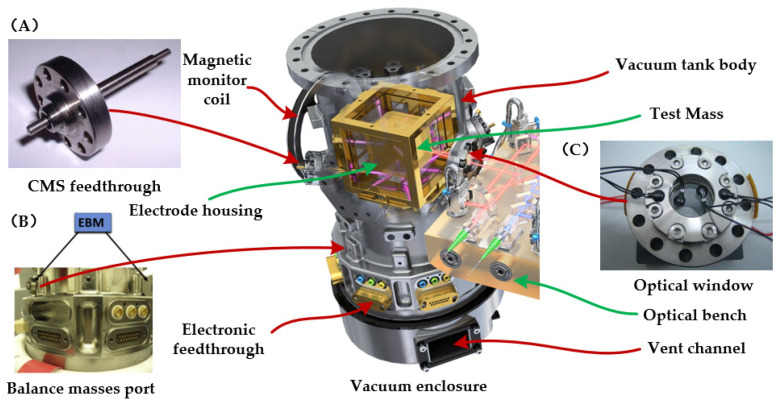
Functional composition of the vacuum maintenance system of LPF [43,57,96,135]. (**A**) Package structure of the charge management system; (**B**) Self-gravity balance masses port and balance mass; (**C**) Optical window mounted on vacuum chamber.

Additionally, the vacuum maintenance system includes various interface units, such as: a mechanical interface for supporting the electrodes housing and CVM components, an electrical interface for connecting to the front-end electronics unit, an optical window on the laser path, a charge management system sealed flange, and a counterweight interface for balancing the self-gravity of the TM, as shown in Figure 15. The optical window primarily addresses the variation in the optical path caused by stress-optical and thermal-optical effects [134,135]. The charge management system flanges are mainly concerned with sealing and integration issues [136]. As discussed in Section 3.4 regarding self-gravity, the local gravitational field around the spacecraft can significantly influence the driving force and torque applied to the TM. This influence can consume system resources, highlighting the importance of balancing all spacecraft components [137]. A well-balanced system minimizes the residual static gravity that requires compensation. To achieve this balance, the LPF implemented a specialized position interface within its inertial sensor vacuum chamber. By adding balance masses [57], the system was able to complete the self-gravity compensation process, although this process needs to be repeated several times.

### 3.7. Summary of the Critical Technologies

Unlike most current space missions, space gravitational wave detection requires an exceptionally high level of technical sophistication due to its complexity and uniqueness, particularly concerning its primary component: the inertial sensor. The inertial sensor is intrinsically linked to both the laser interferometry system and the drag-free control system. Together, these components form a highly complex and precise measurement system, working in unison to enable the high-precision detection of gravitational waves.

On one hand, the inertial sensor provides a high-precision inertial reference to the laser interferometry system, allowing it to accurately measure the distance changes between the TMs. On the other hand, the inertial sensor’s precise measurements help the drag-free control system maintain high-precision control of the satellite platform. This significantly enhances the space gravitational wave detector’s resistance to interference, ensuring stable operation in the space environment. Furthermore, the laser interferometry system can also measure the pose changes of the TMs along the sensitive axis with greater accuracy, feeding this information back to the drag-free control system, thereby enabling the spacecraft to maintain a higher level of drag-free flight precision.

Thanks to a variety of research advancements and technological developments represented by the LISA program since the early 1990s—particularly the LPF mission—significant progress has been made in inertial sensor technologies, along with extensive on-orbit expertise. Numerous functional units and innovative designs have been developed to meet the ultra-low residual acceleration noise requirements for the TMs. This progress has paved the way for the continued advancements in gravitational wave detection missions, and established the following basic technological routes:

1. **Cubic Test Masses:** Made from an Au/Pt alloy, recognized for its high density and low susceptibility to external forces, these serve as the fundamental configuration for the inertial reference.
2. **Molybdenum Electrode Housing:** The molybdenum electrode housing offers exceptional machinability, integration accuracy, and stability. Capacitive sensor-based measurement, along with drive and control methods for the test masses, remain the most common and effective approaches.
3. **Hierarchical Releasing Strategy:** This strategy, which utilizes both caging and releasing mechanisms, ensures the stable locking of the TM prior to orbital entry, while facilitating a precise, ultra-low-speed release once in orbit.
4. **Charge Management System:** A charge management system based on the UV photoelectric effect remains the best method for achieving precise control over the TM’s charge.
5. **Vacuum Maintenance System:** Various interface configurations are employed to provide the structural basis for integrating the inertial sensors, while maintaining an ultra-high vacuum.

Additionally, due to the critical role of the inertial sensor and the challenges of space missions, extensive ground testing is essential. Such testing should aim to focus on understanding the noise characteristics in the space environment, identifying and eliminating the most significant sources of interference, providing model characteristics within budgetary constraints, and defining the upper limits of disturbance forces affecting the inertial sensor. To support these efforts, a specialized torsion pendulum device is required. Detailed discussions on this testing approach are beyond the scope of this article, and readers are referred to the relevant literature for further information.

## 4. Future Trends and Applications of the Inertial Sensor

With the rapid development of space gravitational wave-detection missions, particularly in the area of inertial sensors, many related technologies have reached new milestones and have been validated in space. However, the future systems designed for space-based gravitational wave detection, which will be more complex and integrated than current research efforts, present significant challenges for inertial sensors. These sensors must not only improve performance at the individual unit level but also undergo rigorous integration testing, all while addressing various engineering constraints. Therefore, the critical technologies at this stage will continue to evolve, with increased attention to detail and thorough testing, particularly in the following areas:

1. **High-Precision Detection and Ground Verification:** Focus on the magnetic susceptibility and residual magnetic moment of the TMs to achieve high-precision detection, supported by comprehensive ground-based verification to ensure the reliability of measurements in space.
2. **Multi-DOF Control for TMs:** The multi-DOF control technology for the TMs, under integrated conditions, still requires further refinement in system stability and resolution. Key areas for improvement include reducing power consumption and enhancing noise suppression.
3. **Releasing Mechanism and Ground Testing:** Further investigation, analysis, and testing of the releasing mechanism and associated ground experimental verification devices are necessary to address challenges such as driver output differences and structural vibrations. Additionally, the dynamic model will also be updated, and the test schemes and release strategies will be optimized for better performance.
4. **UV-Based Charge Management Systems:** UV light technology is becoming a viable option for charge management systems due to its lower power consumption, more stable performance, compact size, and reduced cost. Additionally, research will continue to focus on improving fiber coupling efficiency, which remains critical for the performance of other subsystems and the overall system.
5. **Comprehensive Environmental Monitoring:** As spacecraft become more integrated and complex, a more comprehensive approach will be required for monitoring and controlling temperature, magnetic fields, electric charge, gravity, and other critical parameters.
6. **Telemetry and Sensor Integration:** A variety of sensors will be introduced for telemetry purposes, enabling the collection of more detailed data and an accurate depiction of the spacecraft’s on-orbit operational state.

Therefore, based on the design of the functional components outlined above, future inertial sensors will become more flexible, focusing on simplification and miniaturization. These designs can be tailored to optimize performance for a range of space missions, allowing further reductions in size and complexity. At the same time, the technology behind each functional unit has been significantly advanced during implementation, enabling broader applications in space scenarios, including:

1. **Gravity field monitoring:** Detecting variations in gravitational fields on Earth or other celestial bodies to study gravity field distribution, geological structures, and celestial movements.
2. **Attitude control of the spacecraft:** Monitoring changes in spacecraft attitude through a feedback control system to enable attitude adjustments and support drag-free flight.
3. **Dynamic performance evaluation of the spacecraft:** Inertial sensors can directly measure the acceleration and angular velocity of spacecraft, enhancing operational efficiency and stability through optimized design.
4. **Spacecraft positioning and navigation:** Real-time monitoring of spacecraft position, velocity, and orientation. This data can be integrated with navigation systems to provide high-precision navigation information.
5. **Constellations maintain and communication:** Conducting scientific experiments that rely on spacecraft attitude control and navigation to maintain precise satellite constellation configurations.
6. **Deep space explorations:** Monitoring environmental conditions in space, such as electromagnetic radiation, solar radiation pressure, and microgravity states, among others.

In summary, inertial sensors designed for gravitational wave detection must meet system requirements that exceed those of previous space missions in terms of both functionality and performance. Despite the substantial investment required to develop the requisite technologies, the resulting engineering applications and scientific advancements will yield significant benefits. We can expect groundbreaking results from future scientific missions equipped with these advanced inertial sensors.

## Figures and Tables

**Table 1:** Features of the caging and releasing mechanism [110].

Mechanism	Function	Interface
Caging	Applying pre-load to lock the TM; Withstand and sustain launch vibrations; Hand over the TM to releasing mechanism with minimal shock.	Hemispheric structure near eight top angles of the TM.
Releasing	Grabbing the TM; Releasing it at a very low speed.	Dedicated pyramidal indents in the centers of the TM −Z and +Z.

## Data Availability

Not applicable.

## List of Acronyms

AC	Alternating Current
ALIA	Advanced Laser Interferometer Antenna
CHAMP	CHAllenging Minisatellite Payload
CVM	Caging Vent Mechanisms
DC	Direct Current
DOF	Degree Of Freedom
ESA	European Space Agency
GEO600	Gravitational Wave Observatory 600
GOCE	Gravity field and steady state Ocean Circulation Explore
GP-B	Gravity Probe-B
GRACE	Gravity Recovery And Climate Experiment
GRACE-FO	GRACE Follow On
KAGRA	Kansai Advanced Gravitational Wave Observatory
LED	Light-Emitting Diode
LISA	Laser Interferometer Space Antenna
LPF	LISA Pathfinder
NASA	National Aeronautics and Space Administration
ONERA	Office National d’Études et de Recherches Aérospatiales
PID	Proportional, Integral and Derivative
PWM	Pulsed Width Modulation
RF	Radio Frequency
TM	Test Mass
UV	Ultraviolet
VIRGO	Virgo Interferometer for Gravitational waves Observatory
